# Frequency of Harmful Behaviors in Patients Who Are Suffering From Substances Abuse

**DOI:** 10.5812/ijhrba.7403

**Published:** 2012-11-26

**Authors:** Naghmeh Mokhber, Negar Asgharipour, Atiolreza Bananaj

**Affiliations:** 1 Department of Psychiatry, Mashhad University of Medical Sciences, Mashhad, IR Iran; 2 Psychiatry and Behavioral Sciences Research Center, Mashhad University of Medical Sciences, Mashhad, IR Iran

**Keywords:** Substance Abuse, Dangerous Behavior, Suicide, Aggression

## Abstract

**Background:**

Drug abuse is a disorder associated with unhealthy pattern of drug consumption and is a widespread social delinquent both in society and at home. There have only been a few studies conducted on the frequency and types of harmful behaviors among drug abusers.

**Objectives:**

The objective of this study is to investigate the frequency of harmful behaviors in people who are suffering from drug abuse.

**Patients and Methods:**

The population was comprised of all the patients with drug abuse disorders hospitalized during 6 month from 1389 - 1390 in adult wards of Ibn - Sina Psychiatric Hospital in Mashhad. Of those patients who had been identified as having drug abuse disorder, based on the initial interview by a psychiatrist and a urine drug screening test, 99 subjects were selected as the available sample and were informed of the study and its objectives. In this descriptive study patient’s consent had been taken. In psychotic cases, legitimate guardian consent has been obtained. Furthermore, demographic questionnaire in order to determine the harmful behaviors has been completed. Comorbid psychiatric diagnosis was recorded by a psychiatrist, according to the patients’ records. SPSS software was used to analyze the results and, descriptive statistics such as frequency tables and inferential statistics including the chi-square test were used.

**Results:**

85 drug abusers (58.5%) have a history of harmful behaviors and 14 persons (14.1%) have not acted out in this manner. Seventy individuals had participated in more than one harmful behavior. The most common harmful behavior has been reported as aggression by force and attack (65.5 %.).

**Conclusions:**

The most common type of harmful behaviors was “aggression by force and attack”. The next most harmful behavior was defined as the threat of suicide. Clearly, drug abuse was a disorder strongly associated with harmful behaviors.

## 1. Background

Drug abuse is a disorder associated with unhealthy pattern of drug consumption and causes social problems for the individual. These problems include a lack of responsibility in home, workplace, school and even legal problems for the individual (1). Co-occurrence of psychiatric disorders and drug abuse can lead to widespread adverse effects as well as a worse prognosis (2). Several studies have shown that psychiatric disorders, especially personality disorders in patients with drug abuse, are highly prevalent (3, 4). Researchers believe that there is an overlap between self-harmful behaviors and drug abuse as shown by the drug-addicted young individuals who have committed suicide or have suicidal thoughts (5). About 90% of people who have attempted suicide have met one or more criteria of a psychiatric disorder. Disorders that are more associated with suicidal attempts include depression, bipolar disorder, schizophrenia, post-traumatic stress disorder and drug/alcohol consumption. Drug/alcohol abuse is considered the second major risk factor, after psychiatric disorders, for suicide (6, 7). Findings of several studies indicate that harmful and unhealthy behaviors are much more likely to be seen in drug abusers (3, 5, 8). As an example, people with drug abuse disorders pose a higher risk for suicide when compared to individuals with a drug/alcohol abuse disorder who are 6 times more likely to report suicide in their lifetime (7). In addition, studies have shown that the number of drugs is more important than the types of drugs in predicting the probability of suicide (3). Many risk factors of suicide are also applicable for drug abuse disorders. A history of suicidal attempts and a depressed mood are also numbered among such factors and it is believed that “the higher the number of risk factors, the higher probability of abuse” (9-11). Aggression, as one of the common behavioral problems, is also regarded as one of the risk factors for suicide in drug abusers. About 70% of individuals who are referred for treatment of addiction have reported involvement in violence in their history (e.g., a physical attack; attacking with guns and giving beatings) (9, 10). Former studies have shown that there is a relationship between drug abuse and suicide risk and violence plays a role in it. Tait et al. (12) noted that individuals who are unable to control their anger are more likely to act impulsively and focus their anger on themselves rather than others. A study done on 6000 adult drug abusers has shown that individuals with a history of aggression (such as attacks resulting in serious injury and assault), were two times more likely to report suicide attempt. These findings were confirmed even after demographic variables control (13). The findings of a study also showed that there is a significant difference among different groups of drug and stimulant abusers in terms of high-risk sexual behavior and self-harm behaviors. Furthermore, the rate of self-harm behavior is higher in stimulant drug abusers than in opium and glass abusers (8). In general, given the overlap of harmful behaviors and drug abuse and the need to identify individuals who are at high risk of suicide, understanding these behaviors and their prevalence among drug and alcohol abusers is crucial. Since few studies have been conducted on frequency of harmful behaviors and their types among drug abusers, the goal of this study is to investigate the frequency of harmful behaviors in people suffering from drug abuse.

## 2. Objectives

This study aims to investigate the frequency of harmful behaviors in people suffering from drug abuse.

## 3. Patients and Methods

In this descriptive study all patients with drug abuse disorders hospitalized in adult wards of Ibn Sina Psychiatric Hospital. In six month duration from december 2010 – June 2011 all patients suffering from drug abuse disorder (99 subjects), based on the initial interview by a psychiatrist and a urine drug screening test. After obtaining informed consent, a demographic and harmful behaviors questionnaire had been filled. Comorbid psychiatric diagnosis was recorded by a psychiatrist, according to the patients’ records. Inclusion criteria were as follows: Patients agreement to participate, no mental retardation, no serious physical disorder or disability.

### 3.1. Demographic Characteristics Questionnaire

The questionnaire used to obtain patient demographic information included items such as age, sex, marital status, occupation, and education.

### 3.2. Harmful Behavior Questionnaire

Contain of seven items covering areas such as suicide, threats of suicide, threat of killing others, aggression by force and attack, attacking others, injury to others and self-harm which the patients completed in the form of yes/no response. Test-retest reliability was estimated to be 0.93. For validation ten psychiatrists were asked to identify the content of the test to be developed.

### 3.3. Statistical Analysis Method

Descriptive statistics such as frequency tables and inferential statistics including chi-square test were used. This study was performed on 99 subjects of whom 81 were male (81.8%) and 18 were females (18.2%). In terms of marital status, 48 subjects (48.5%) were single, 26 (26.3%) were married and 25 (25.3%) were divorced. Considering axis1 diagnosis, there were 40 patients with schizophrenia, 55 with bipolar disorder and 4 with schizoaffective disorder. The average age of these individuals was about 31 years (between 18 to maximum of 54 years). Education level of patients was as follows: 38 patients had primary school diploma (38.4%); 30 patients had guidance school up to high school diploma (30.3%) and 31 patients had high-school diploma or higher (31.3%). The most commonly used drug in subjects was alcohol (48.5%). Level of education in the study group of individuals with harmful behavior is as follows: 31 patients had maximum primary school diploma (36.5%); 27 patients had guidance school up to high school diploma (31.8%) and 27 patients had high-school diploma or higher education.

## 4. Results

**Figure 1:**
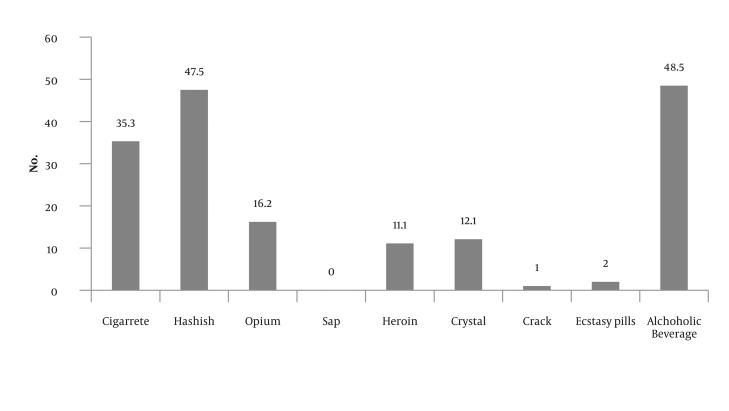
Frequency Distribution of Suicide Methods among Patients with Substance Abuse

**Figure 2:**
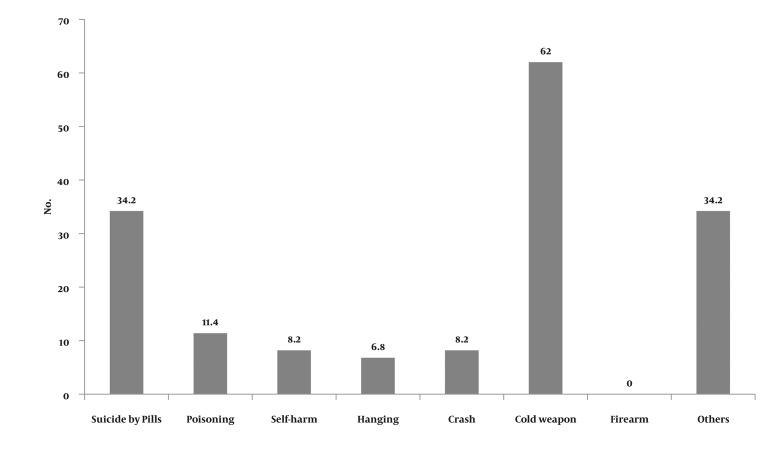
Frequency Distribution of the Materials Used by the Patients with Substance Abuse

Analysis demonstrates (Table 1 and Table 2) that 85 drug abusers (58.5%) have had harmful behaviors and 14 cases (14.1%) had not shown such behaviors. Seventy people showed more than one harmful behavior. The most common harmful behavior has been aggression by force and attack (65.5 %.). Among the subjects, 35 (53.3%) had attempted suicide and the most common suicide method was the use of cold weapons (22.2%). There were 67 males (67.7%) and 18 females (18.2%) among people who had exhibited harmful behavior (Figure 1). The most common substances used were alcohol and cannabis respectively (Figure 2). The effects of gender, marital status and education on committing harmful behaviors, which were studied based on chi-square test, were not significant (P > 0.05). To investigate the effect of age on harmful behaviors, Mann-Whitney Test was used according to which the effect of age on the harmful behavior was not significant (P > 0.05).

**Table 1:** Demographic Characteristics of Patients with Substance Abuse

	Patients With Substance Abuse, No. (%) (n = 99)	Patients With Harmful/Malignant Behavior %85, No. (%)
**Gender**		
Female	18 (18.2)	18 (21)
Male	81 (81.8)	67 (79)
**Marital status **		
Single	48 (48.5)	40 (47.1)
Married	26 (26.3)	22 (27.1)
Divorced	25 (25.3)	23 (25.9)
**Age, y**		
18 - 25	27 (27.3)	22 (25.9)
26 - 35	44 (44.4)	38 (44.7)
> 35	28 (28.3)	25 (29.4)
**Education**		
Up to primary school diploma	38 (38.4)	31 (36.5)
Guidance school up to high school diploma	30 (30.3)	27 (31.8)
High-school diploma and higher	31 (31.3)	27 (31.8)

**Table 2:** Frequency of Harmful Behaviors Among Patients with Substance Abuse (n = 99)

	No Harmful Behavior	Suicide	Threats of Suicide	Threats of Killing Others	Aggression with Force and Attack	Against Others	Injuring Others	Self-Harm
**Total number of individuals, No.**	14	35	51	41	65	9	33	42

## 5. Discussion

This study was conducted to investigate the harmful behaviors in drug abusers. Of 99 cases who were suffering from drug abuse, 85 individuals had shown some kind of harmful behavior. This may not be generalizable to the entire population who use different kinds of drugs. That is because the study had been conducted on those who were referred to psychiatric hospitals. Therefore, it is likely that the severity of the disorder and associated diseases be more causal than of the whole of drug abusers in the community. In previous studies, one of the common causes the drug-dependent individuals were referred to hospitals has been associating mental disorders such as personality problems and mood disorders (2). Many drug abusers are also referred to health centers due to drug toxicity. However the number of individuals in this study was not high because the hospital where the study was conducted lacked a toxicology department. The number of male was much higher than female. In the former studies, most cases with drug abuse were males, although the rates of using various drugs in women are also on the rise (2, 14, 15). In addition, most subjects were living alone (unmarried or divorced). This fact has been proved in previous studies; particularly the number of divorced individuals verifies their problems in interpersonal relations. It is interesting to note that no significant difference was found in the incidence of harmful behaviors in women and men. Some studies have shown that harmful behaviors have higher frequency in men (2, 7). Since these types of studies had been conducted in different communities, they differed from the present study in terms of the population. Furthermore, the present study is limited to psychiatric hospital cases. Therefore, the statistical difference is justifiable with regard to this matter. None of the variables, education and marital status, were identified as being effective in the incidence of harmful behaviors. But a study conducted at Shiraz University of Medical Sciences, Iran has shown that the incidence of harmful behaviors is more common in people with lower educational levels (16). Our study had been performed on those who lacked comorbid axis I psychiatric disorders. Thus, the design of Shiraz University investigation is different from the current study in terms of the overall structure and methodology. Few studies reported more prevalence of harmful behaviors in unmarried subjects than in married ones, have emphasized social adaptation techniques. The present study had not considered this aspect of the subject’s behaviors. Considering the incidence of harmful behaviors in this study the most common type was “aggression by force and attack”. Subsequently, “the suicidal threat” was the other important factor. Various reports have been presented in previous studies, for example in the study performed by McCloskey et al. (17), similar to our study, aggressive behavior had been the most prevalent indicator. However, another study showed a higher incidence of suicide and self-harms (18). The afore-mentioned study differs from the present study in that it had been done with people with personality disorders. In addition, according to Iranian cultural and religious norms, a lower incidence of suicide is reasonable. Considering the methods of suicide, the most common methods had been the use of cold weapons and pills, respectively. Given that most of the subjects were male and use of weapons was more common than pills or poison in men, the above results were predictable. None of the subjects had used firearms, perhaps due to their unavailability. However, one should also consider the fact that this method is very effective and dangerous; and death occurs so quickly that there would be no chance of saving the patient or sending him/her to a psychiatric hospital. According to our findings, the most commonly used drugs were alcohol and cannabis respectively. Use of alcohol in other studies, as the most commonly abused substance, had been proven (8, 19). Since the study was conducted on the patients of hospital wards with psychiatric disorders; therefore, the use of alcohol, as the most commonly used drug, might not be representative of the whole community. Interestingly, associated psychiatric disorders were prevalent in these patients, with bipolar disorder as most common disorder (type 1 and 2) and schizophrenia as the second. Since the research was conducted in a psychiatric hospital, higher prevalence of psychiatric disorders was understandable. Using various materials that had already been proved in mood disorders (2) has also been approved in the present study. Since our study is among the first demographic research of drug abuse and harmful behaviors in Iran, it can be used as a guide for other studies. Nevertheless, this study also had some limitations. Since this study was conducted on hospitalized patients, generalization might be problematic. Also bigger sample size in longer period is recommended in order to obtain reliable information. Some patients might not record their substance use and therefore this study might not include all patients suffering from substance abuse disorder. Investigation of specific personality disorders as well as individuals’ methods of compliance could yield valuable information. Conducting the study in different centers and studying different demographic bases can help us in achieving more reliable results.
